# Crystal Structure of Isoform CBd of the Basic Phospholipase A_2_ Subunit of Crotoxin: Description of the Structural Framework of CB for Interaction with Protein Targets

**DOI:** 10.3390/molecules25225290

**Published:** 2020-11-13

**Authors:** Dorota Nemecz, Maciej Ostrowski, Marc Ravatin, Frederick Saul, Grazyna Faure

**Affiliations:** 1 Institut Pasteur, Récepteurs-Canaux, CNRS UMR 3571, Département de Neuroscience, 25, rue du Dr. Roux, F-75015 Paris, France; nemecz@umk.pl (D.N.); maciejost@umk.pl (M.O.); marc.ravatin@pasteur.fr (M.R.); 2 Biochemistry Department, Faculty of Biological and Veterinary Sciences, Nicolaus Copernicus University, 87-100 Torun, Poland; 3 Sanofi R&D, Integrated Drug Discovery-High Content Biology, 94400 Vitry-sur-Seine, France; 4 Institut Pasteur, Plateforme de Cristallographie-C2RT, CNRS UMR 3528, 75015 Paris, France; frederick.saul@pasteur.fr

**Keywords:** phospholipase A_2_ isoforms, crystal structure, protein-protein interaction, anticoagulant binding site, CFTR binding interface

## Abstract

Crotoxin, from the venom of the South American rattlesnake *Crotalus durissus terrificus,* is a potent heterodimeric presynaptic β-neurotoxin that exists in individual snake venom as a mixture of isoforms of a basic phospholipase A_2_ (PLA_2_) subunit (CBa_2_, CBb, CBc, and CBd) and acidic subunit (CA_1–4_). Specific natural mutations in CB isoforms are implicated in functional differences between crotoxin isoforms. The three-dimensional structure of two individual CB isoforms (CBa_2_, CBc), and one isoform in a crotoxin (CA_2_CBb) complex, have been previously reported. This study concerns CBd, which by interaction with various protein targets exhibits many physiological or pharmacological functions. It binds with high affinity to presynaptic receptors showing neurotoxicity, but also interacts with human coagulation factor Xa (hFXa), exhibiting anticoagulant effect, and acts as a positive allosteric modulator and corrector of mutated chloride channel, cystic fibrosis transmembrane conductance regulator (CFTR), implicated in cystic fibrosis. Thus, CBd represents a novel family of agents that have potential in identifying new drug leads related to anticoagulant and anti-cystic fibrosis function. We determined here the X-ray structure of CBd and compare it with the three other natural isoforms of CB. The structural role of specific amino acid variations between CB isoforms are analyzed and the structural framework of CB for interaction with protein targets is described.

## 1. Introduction

Crotoxin from the venom of the South American rattlesnake *Crotalus durissus terrificus,* is a potent β-neurotoxin with phospholipase A_2_ (PLA_2_) activity. It is a heterodimeric protein complex formed by the non-covalent association of a basic PLA_2_ subunit of low toxicity (CB), and a nontoxic acidic subunit (CA) devoid of catalytic activity that potentiates the toxic effect of CB, by enabling the toxin to reach the specific crotoxin receptor at the neuromuscular junction [1]. The CA and CB subunits are present in individual snake venom as a mixture of isoforms (CA_1–4_ and CBa_2_, CBb, CBc, CBd) that can form up to 16 natural crotoxin complexes with different biochemical and pharmacological properties [2,3,4]. Based on these differences, two classes of crotoxin (class I and II) were determined [5]. Class I, composed of CBb, CBc, CBd with any of the CA isoforms, contains very stable complexes (half-life 10–20 min, K_d_ = 4.5 nM) of high toxicity (these isoforms block neuromuscular transmission of chick biventer cervicis preparations more efficiently than weakly toxic isoforms) and low PLA_2_ activity. Class II, composed of CBa_2_ with any of the CA isoforms, contains less stable complexes (half-life about 1 min, K_d_ = 25 nM) of low toxicity and high enzymatic activity [5].

Crotoxin acts primarily at the presynaptic level and induces complete failure of neuromuscular transmission by interacting with specific neuronal targets [6,7,8]. In addition to neurotoxicity, crotoxin exhibits other pharmacological and biological properties, including antiviral [9], cytotoxic (against tumor cells) [10,11,12], anti-inflammatory [13,14], immunomodulatory (via dendritic cells) [15], or analgesic [16] effects. It can also induce calcium-dependent glutamate release via N and P/Q calcium channels [17]. The CB PLA_2_ subunit purified from a crotoxin complex, may also act independently on different biological systems: (i) it blocks neuromuscular transmission by binding to presynaptic receptors (the neuronal CB target is different from that of the crotoxin receptor, and 7-fold higher doses of CB, with respect to class I crotoxin complexes, are needed to achieve neurotoxicity) [5,7,18]; (ii) it exhibits anticoagulant properties through direct interaction with human coagulation factor Xa (hFXa), leading to inhibition of the prothrombinase complex required for efficient production of thrombin [19]; (iii) it forms a complex with the ΔF508-NBD1 and WT-NBD1 (nucleotide-binding domain 1) of the cystic fibrosis transmembrane conductance regulator (CFTR), and acts as a positive allosteric modulator and corrector of the mutated chloride channel [20]; (iv) it interacts with *Gloeobacter* ligand-gated ion channel (GLIC), a member of the pentameric ligand-gated ion channel (pLGIC) family, as a negative allosteric modulator [21]; and (v) it forms a complex with the natural crotoxin inhibitor from *Crotalus* serum (CICS), which neutralizes its neurotoxicity and enzymatic activity [22,23,24]. Crotoxin and its CB PLA_2_ subunit are of interest because of potential therapeutic perspectives, leading to active investigation of their mechanism of interaction with various biological targets at the molecular and cellular level [25].

We previously solved the crystal structure of the class I crotoxin isoform CA_2_CBb and identified the binding interface between the two subunits, showing the crucial role of residue His1 in the formation of the more stable interface between Trp31 and Trp70 of CBb, with Asp99 and Asp89 of CA_2_ (amino acids are numbered according to Renetseder [26]) [27]. Moreover, the crystal structure of CBa_2_ (CB2, with Ser in position 1) and CBc (CB1, with His in position 1) in a tetrameric complex without the CA subunit, revealed conformational differences between these two isoforms [28]. A comparison of the individual CB isoform crystal structures and the correlation of their differences with the CA_2_CBb complex, shows how the structural changes within CB contribute to the functional differences between the two classes of crotoxin complexes [27].

In the present work, we report the crystal structure of the most abundant CB isoform of crotoxin, CBd. It is now possible to compare the crystal structure and function of the four natural CB isoforms. The structural role of specific amino acid variations between CB isoforms are discussed herein, and the structural framework of CB for interaction with protein targets is described.

## 2. Results

### 2.1. Three-Dimensional Structure of CBd Isoform-General Description

**Figure 1:**
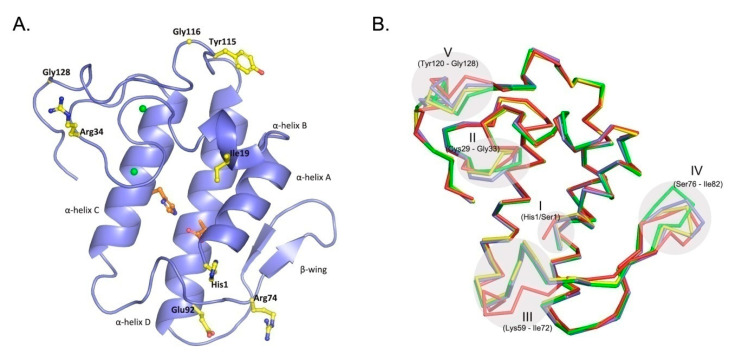
Overall three-dimensional structure of the CBd isoform. (**A**) Crystal structure of CBd, with distinguishing residues at positions 1, 19, 34, 74, 92, 115, 116, and 128 shown in yellow stick representation. The α-helices A–D and the β-wing are labeled and the His48/Asp99 pair of the catalytic site is shown in red. (**B**) Superposition of the α-carbon backbones of the four natural CB isoforms which have been crystallographically resolved: CBa_2_ (pdb 2QOG, polypeptide chain A) in red, CBb (pdb 3R0L, polypeptide chain D) in green, CBc (pdb 2QOG, polypeptide chain B) in yellow and CBd (this study, pdb 6TMY) in blue. Five conformationally variable or flexible regions are indicated (see text).

The crystal structure of CBd (Figure 1A) was determined at 1.8 Å resolution. The asymmetric unit of the crystal contains six individual CBd molecules. A summary of the crystallographic parameters and data, as well as refinement statistics, are shown in Table 1.

**Figure 2:**
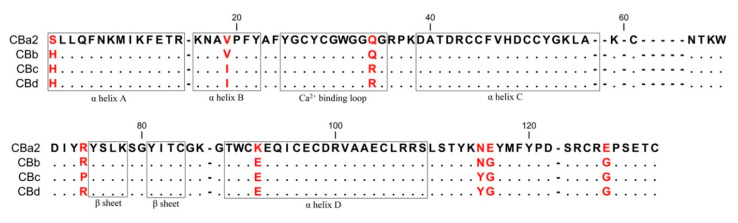
Alignment of amino acid sequences of the four natural CB isoforms. Distinguishing residues of the isoforms are indicated in red. Sequences are from the GenBank/NCBI database: CBa_2_ (P24027.1), CBb (P0CG56.1), CBc (P62022.1) and the sequence of CBd from this study. Residues are numbered according to Renetseder et al. [26].

As expected, the structure of CBd is very similar to the other natural CB isoforms (CBa_2_, CBb, and CBc) and to other group IIA sPLA_2_ (EC 3.1.1.4). This canonical structure contains an *N*-terminal α-helix (A) followed by a short helix (B), a calcium-binding loop, a long α-helix (C), an anti-parallel two-stranded beta sheet (the β-wing), a long α-helix (D) anti-parallel to helix C, and a C-terminal extension (Figure 1A). The overall structure is stabilized by seven disulfide bonds. However, comparison of the crystal structures of the four natural isoforms of CB revealed five conformationally variable or more flexible regions (Figure 1B). Region I applies to a single residue in position 1 (His/Ser, depending on the class of crotoxin) at the *N*-terminal of α-helix A. Region II (residues 29–33) is the canonical calcium-binding loop. Region III (residues 59–72) is located in a loop between α-helix C and the β-wing. Region IV (residues 76–82) is a part of the β-wing. Region V (residues 120–128) occurs at the C-terminal extension and contains a Gly/Glu variation at position 128 (Gly in CBb, CBc, CBd; Glu in CBa_2_). The crystal structure also revealed the amino acid sequence of CBd, including specific distinguishing single-residue variations (Figure 2). Amino acid residues are numbered according to Renetseder [26].

Two sodium ions were observed per CBd molecule in the crystal structure presented here. One Na^+^ binds in the canonical sPLA_2_ calcium-binding loop, making hydrogen bonding contacts with the carbonyl atoms of the polypeptide chain at positions Tyr28, Gly30, and Gly32, and a bidentate hydrogen bond with the side chain of Asp49. This pattern of interactions in the calcium-binding loop is also observed in the crystal structure of isoform CBb [27] and is a common feature in sPLA_2_ structures [29]. A second Na^+^ makes interactions with the backbone carbonyl oxygen of Phe24, Gly26, and Tyr120 as in the crystal structure of the heterodimeric CA_2_CBb crotoxin complex [27]. This second Na^+^ binding is weaker and lacks full coordination in two of the six individual CBd molecules in the asymmetric unit of the crystal. All six CBd molecules in the asymmetric unit contain PEG 400 (present in the crystallization solution) in the interfacial binding region near the canonical calcium-binding site.

### 2.2. Oligomeric Assembly of CBd Isoform

**Figure 3:**
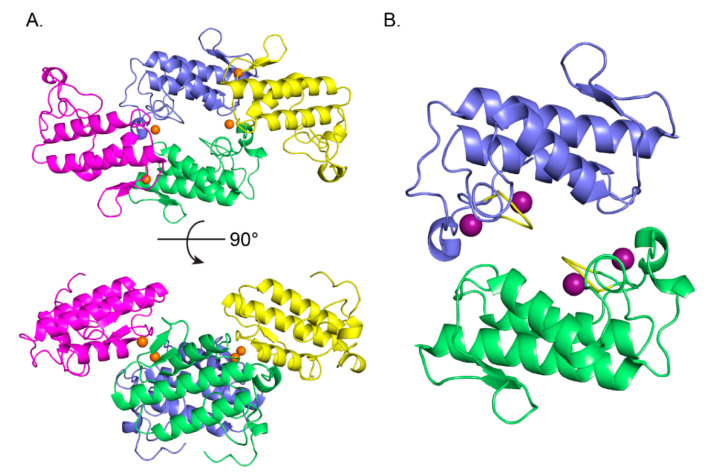
Oligomeric assembly of the CBd isoform. (**A**) Tetrameric quaternary organization of CBd (polypeptide chains A in green, E in blue, B in magenta, C in yellow). Chloride ions are shown as orange spheres. (**B**) Structure of the central CBd-CBd dimer (polypeptide chains A,E). Two Na^+^ binding sites in each monomer are shown as purple spheres and the canonical calcium-binding loop is shown in yellow.

Analysis of the protein interfaces in the crystal structure of CBd using the PDBePISA web-based interactive tool [30] revealed two probable tetrameric quaternary assemblies of isoform CBd. These assemblies are composed of polypeptide chains A,E,B,C in a non-crystallographic tetramer (Figure 3A), and chains D, F in a crystallographic tetramer (2D, 2F, not shown). The two tetramers are very similar, with an rms deviation of 1.12 Å over all alpha-carbons of the superimposed structures. The total buried surface area in the AEBC and 2D2F tetramers is 9730 Å^2^ and 9070 Å^2^, respectively. The tetrameric assembly of CBd can be considered as a central dimer (A,E or D,D) associated with two peripheral monomers (B,C or F,F), respectively (Figure 3B). The most extensive contacts in the central dimeric interface are made by residues His1, Leu2, Leu3, Ile19, Phe24, Trp31, Gly32, Ala56, Lys59, Asn67, Lys69, Trp70, Phe119, Tyr120, Pro121, Asp122, and Arg127. Interestingly, the central dimer interface overlaps with the sPLA_2_ interfacial-binding surface [31] and the CA-binding interface of CBb in the CA_2_CBb crotoxin complex [27].

**Figure 4:**
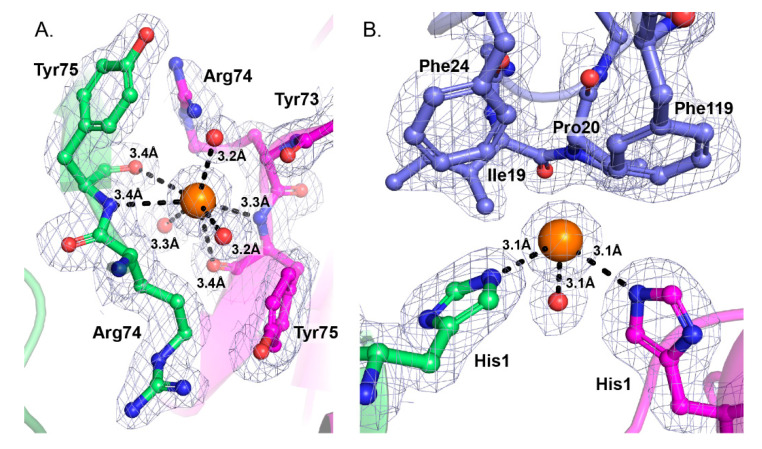
Interactions of chloride ions contribute to the stability of the tetrameric quaternary assembly. (**A**) Cl^−^ binds to the polypeptide chains at Tyr75 from two adjacent monomers (chains A and B). Chains are colored as in Figure 3. (**B**) Cl^−^ binds to the side chains of His1 from the two adjacent CB monomers (chains A and B). Additional bonds are made with nearby water molecules. Residues within 4 Å are labeled and shown as sticks. The bond distances are indicated. Chloride ions are represented as orange spheres and coordinating water molecules are shown as red spheres. The electron density map is contoured at the 1.0 sigma level.

Extensive crystal contacts are made between a central and peripheral monomer in the tetrameric assembly, between the β-wing and α-helix A. Chloride ions in the structures reinforce these contacts. One Cl^−^ binding site is located between the β-wings of two adjacent monomers, and makes binding interactions with the polypeptide chains of Tyr75 from the two monomers. (Figure 4A). A second chloride site is also coordinated by binding interactions with the side chains of His1 from a central and a peripheral monomer of the tetrameric assembly (chains A,B) (Figure 4B). A complementary hydrophobic interface from a third monomer (chain E) stabilizes interactions in region of the second Cl^−^ binding site. Residues Ile19, Pro20, Phe24, Phe119 are within 4 Å of the Cl^−^ binding site (Figure 4B). Other nearby hydrophobic residues include Met118 from chain E and Trp70 from chain B.

Additional crystal contacts are made between the central and peripheral monomers principally through interactions of Arg14 with either Glu12 or Phe11, and interactions of the tip of the β-wing.

## 3. Discussion

### 3.1. Residue Variations in CB Isoforms Distinguish between Class I and II Crotoxin Complexes

Classification of crotoxin complexes was made according to their stability, toxicity, and enzymatic activity, which depend solely on the isoform of the CB subunit in the complex [5]. Isoform CBd was previously classified to class I since it possesses biological properties similar to CBb and CBc in complex with CA [5]. Class I forms very stable and toxic crotoxin complexes with low enzymatic activity. The sequence determination of CBd from the crystal structure, and its comparison to the other CB isoforms clearly reveal four residues that distinguish between two classes (I and II) of crotoxin complexes. These are His1Ser, Glu92Lys, Gly116Glu, Gly128Glu in CBd, CBc, CBb (class I) and CBa_2_ (class II), respectively (Table 2).

His1Ser: The presence of His at position 1 stabilizes the *N*-terminal region through the formation of a hydrogen bond network. This network originates from a hydrogen bond between the πN of His1 and the backbone amine of Leu3. This interaction stabilizes the *N*-terminal amine and allows for formation of two hydrogen bonds with the side chain oxygen of Gln4 and the carbonyl backbone of Asp71. Gln4 is then locked in place and thereby forms two respective hydrogen bonds between its side chain amide atoms and the main chain amide of Tyr73. Additional hydrogen bonds are observed between the backbone carbonyl of Lys69 and the backbone amine of Leu2. These arrangements create the observed conformational structure in region III for CBb, CBc, and CBd, and facilitate the direct interaction of Trp70 with Asp89 of the acidic CA subunit, leading to increased stability of the crotoxin complexes [27] (from K_d_ = 28 nM for CBa_2_ to K_d_ = 5 nM for CBb, CBc, and CBd [5]).

Glu92Lys: In CBa_2_, which lacks *N*-terminal stability through the presence of Ser1, the Lys92 could make a hydrogen bond to the side chain of Asp71 leading to further destabilization of the *N*-terminal and a conformational change in region III with respect to class I CB isoforms. In class I complexes the presence of Glu92 does not interfere with the above-mentioned hydrogen bond network.

Gly116Glu, Gly128Glu: The C-terminal region of CB, does not participate in the formation of the crotoxin complex [27], but might be involved in the neurotoxic activity of crotoxin [32]. This region in CBd, CBc, and CBb isoforms (of class I) is strongly positively charged. The presence of two negatively charged residues: Glu116 and Glu128 in CBa_2_ (instead of Gly116 and 128) results in a significant change in surface charge of the molecule, which may affect the binding to specific receptors (such as CAPT-48 from *Torpedo marmorata* [8]) and the overall toxicity of the crotoxin.

### 3.2. Role of the Differences in Amino Acid Sequences between CBd, CBc, and CBb on the Properties of Class I Crotoxin Complexes

Our previous studies showed that the biological properties of CBb, CBc, and CBd, and the respective crotoxin complexes, are very similar [5]. The amino acid sequences of the CB isoforms of class I are also highly similar. CBd differs from CBc by only one amino acid (Arg74Pro, respectively) and by three amino acids from CBb (Ile19Val, Arg34Gln, and Tyr115Asn, respectively). Comparative analysis of the crystal structures of all four CB isoforms suggests that these differences do not affect the catalytic activity of CB or the toxicity of crotoxin; however, there may be slight effects upon the interaction of CB with the CA subunit. Specific evaluation of each position is as follows:

Ile19Val: In the region around position 19 (Ile/Val) the structures of all isoforms are similar. The side chain is solvent exposed and centrally located in the CA binding interface. Steric bulk differences between Ile and Val could have a small effect on binding, but there is no observed correlation with toxicity [27].

Arg34Gln: The Arg side chain at position 34 appears to be highly flexible in the resolved crystal structures, whereas the main chain conformation is similar in all CB isoforms. Since this solvent-exposed residue does not participate in any binding, the mutation to Gln (in CBb) does not appear to play any significant role in the functional properties of CB and crotoxin complexes. However, changes in the intrinsic properties of CB through the presence of Arg and the addition of a positive charge on the protein surface may have other significant consequences.

Arg74Pro: Comparison of the available CB structures, in which only CBc contains Pro at position 74, shows the two adjacent Tyr residues (at position 73, 75), along with the main chain at position 74, in the same conformation in all isoforms. However the presence of Pro74 in CBc, instead of Arg, could lead to reduced flexibility of the polypeptide chain in this region and could lead to a distinct change in properties between the two protein variants. 

Tyr115Asn: In the crystal structure of the CA_2_CBb complex [27], Asn115 (in CBb) is solvent exposed and situated near Gly35 of the CA subunit α-chain. However, the distance between these two residues (greater than 6 Å) is too large to generate any significant interaction. In contrast, Tyr115 (in CBc and CBd) could form a hydrogen bond with the polypeptide chain or the side chain of Gln34 of the CA α-chain. Indeed, Fortes-Dias et al. confirmed the importance of Tyr115 in protein-protein interactions [33]. They showed that the region of CBc containing Tyr115 plays an important role in CICS/CNF binding (a natural crotoxin inhibitor from snake blood). According to these studies, even 10 molar excess of CA did not lead to dissociation of the inhibitor bound to this region. In the CBa_2_ isoform, in which Asn115 is present, this region did not participate in CICS binding [33].

### 3.3. The Natural CA-Binding Domain on CB Serves as the Structural Framework for Interaction with Other Biological Targets: Human Coagulation FXa and Human CFTR

It was previously shown that the CB subunit of crotoxin, acting independently of the crotoxin complex can exhibit various pathophysiological and pharmacological functions [7,19,20]. We showed that at presynaptic membranes, the isolated CB can recognize similar protein receptors, such as monomeric ammodytoxin (Atx). since CB was able to completely inhibit the binding activity of radio-iodinated ammodytoxin [7]. Several other protein targets, including human coagulation FXa and human CFTR, were identified and characterized as a target for this PLA_2_, showing its anticoagulant or anti-cystic fibrosis activity [19,20]. All of these previously studied mechanisms are independent of catalytic activity of PLA_2_ and are based on direct protein-protein interaction. CB can affect hemostasis by binding to human FXa, and can inhibit formation of the prothrombinase complex required for efficient production of thrombin by the non-enzymatic, phospholipid-independent, anticoagulant mode of action [19]. More recently, we showed that CB forms a high nanomolar affinity complex with NBD1, as well as its mutated form ΔF508-NBD1 of CFTR [20]. CB could act as a positive allosteric modulator increasing Cl^−^ channel current of CFTR and as corrector of the ΔF508-CFTR mutant implicated in cystic fibrosis disorder [20].

**Figure 5:**
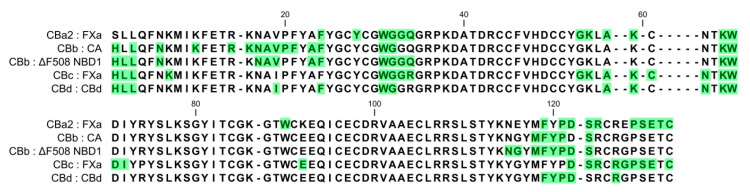
Sequences of CB isoforms showing the key residues in the interface regions of the specified complexes. Green-shadowed characters denote residues at the interface of CBa_2_/hFXa [19], CBb/CA [27], CBb/ΔF508-NBD1 [20] of CFTR, CBc/hFXa [19], and CBd/CBd [this study].

**Figure 6:**
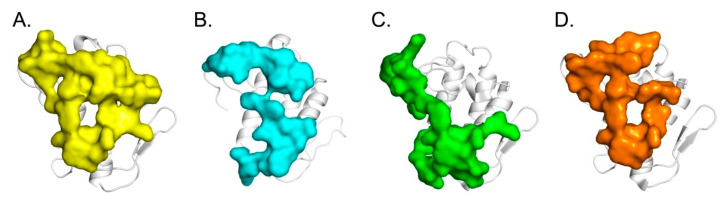
Functional sites of CB, a basal platform for protein-protein interaction. (**A**) CA-binding site identified by crystallography (in yellow) [27], (**B**) CBd-CBd dimer site identified by crystallography (present study, in blue), (**C**) hFXa-binding site generated by molecular docking (in green) [19] and (**D**) ΔF508-NBD1-binding site identified by molecular docking and HDX-MS experiments (in orange) [20].

The three-dimensional structure of the crotoxin complex revealed the nature of the binding interface between the CA and CB subunits and allowed us to identify key amino acid residues responsible for significant differences in the stability, toxicity, and enzymatic activity of the two classes of crotoxin complexes [27]. Molecular docking analysis supported by hydrogen deuterium exchange mass spectrometry (HDX-MS) and surface plasmon resonance (SPR) competition studies [20]. showed that regions of CB participating in the interaction with ΔF508-NBD1 overlap with those involved in forming the natural CA-CB complex (residues His1, Leu3, Asn6, Asn17, Ala18, Val19, Ala23, Phe24, Trp31, Gly32, Lys69, Trp70, Met118, Phe119, Tyr120, Pro121, Ser124) [20]. Similar studies using SPR showed that the hFXa-binding site partially overlaps with the CA-binding site of CB (residues His1, Leu3, Phe24, Trp31, Gly32, Lys69, Trp70, Phe119, Pro121, Ser124) [19,34,35]. Interestingly, it was shown by analytical ultracentrifugation that CA and hFXa prevent oligomerization of CB [36]. Interfacial binding surface (IBS) residues of CB (Leu2, Leu3, Ile19, Phe24 and Lys69, identified by homology with Atx C [37], overlap with the interface of complexes CA-CB and CB-hFXa, but also CB-NBD1 and CB-CB. Protein targets (CA, hFXa, or NBD1) therefore prevent CB contact with phospholipids. The CA-, hFXa-, and ΔF508-NBD1-binding sites of CB are shown in Figure 5 and Figure 6. Three regions containing six conserved amino acid residues appear to play an important role in protein-protein interactions. These are α-helix A (His1 and Leu3), the calcium-binding loop (Trp31 and Gly32), and a loop preceding the β-wing (Lys69 and Trp70). Additionally, the C-terminal region of CB, despite the lack of conservation, also actively participates in the aforementioned interactions. The side-chains of these residues are exposed on the surface of the CB molecule, presenting favorable orientations for interaction with CFTR or hFXa. Interestingly, our previous study showed that hFXa-binding site of PLA_2_ (Atx A and C) partly overlaps with the CaM-binding site [38]. Characterization of this basal CB pharmacological platform offers promising perspectives for structure-based design of new anti-cystic fibrosis and anticoagulant therapeutics.

### 3.4. Pathophysiological Relevance of Dimer of CBd

The CBd crystal structure was resolved as a homogenous oligomer of four monomers, which form a central dimer connected with two peripheral molecules. A tetrameric structure was also obtained by Salvador-Marchi and colleagues [28]; however, in this case it contained two heterodimers each composed of CB1 (CBc) and CB2 (CBa_2_) isoforms [28]. The interface between the heterodimers, the CB1 (CBc) homodimer, resembles the central dimer described in the present structure. The physiological or pathophysiological relevance of the oligomeric formation of CB subunits is still unclear. In rattlesnake venom, all CB isoforms are present in complex with the CA subunit, which prevents oligomerization of CB. The region of CB participating in the formation of the central dimer overlaps with the region involved in forming the natural CA-CB complex (Figure 5 and Figure 6). Therefore, the formation of oligomer structures does not seem to be a natural conformation and may be due to crystallization conditions, as variations in pH have been shown to affect the CB oligomerization state [39].

However, we still do not know the mechanism of crotoxin-receptor interactions at the atomic level. Formation of the ternary complex, CA-CB-Receptor, and dissociation of CA at equilibrium (with only CB attached to the receptor) have been previously proposed by biochemical and SPR studies [8].

Similarly to the CA-CB interaction, the human coagulation factor Xa also prevents oligomerization of CB, showing the importance of the monomeric state of this PLA_2_ for anticoagulant action [36]. According to this data, the formation of CB oligomers does not seem to be a physiological process. However, the CB dimer could create more accessible surface areas for interaction with specific PLA_2_-receptors by increasing the number of binding sites [28,38,40]. According to this hypothesis, tetrameric or dimeric configurations may play an important role in neurotoxicity or other functions of human inflammatory sPLA_2_GIIA. Moreover, in pathological conditions, where high concentrations of sPLA_2_ have been detected [41,42,43], formation of sPLA_2_ oligomers could be expected. Additional in vivo studies are needed to explain the role of oligomerization of sPLA_2_ in pathophysiological processes.

## 4. Materials and Methods

### 4.1. Purification of Isoform CBd 

Four CB isoforms were isolated from the crude venom of *Crotalus durissus terrificus,* as described previously [3]. In brief, whole venom was fractionated on a Sephadex G-75 column and the major peak of crotoxin (a mixture of crotoxin isoforms) was isolated. The CA and CB subunits were separated by anion exchange chromatography in 6 M urea and dialyzed. The isoforms of CA subunit were purified on a Mono Q column and the isoforms of CB were purified on a Mono S column [3] followed by reversed phase chromatography for each isoform on a Vydac C4 column [44]. The purity of the CB isoforms was confirmed by electrospray mass spectrometry [45], or non-denaturing electrophoresis after reconstitution of the crotoxin complexes (one CB isoform with one CA isoform) using 20% homogenous PhastGel [3]. Protein concentration was determined spectrophotometrically as described [3]. A single, homogenous CBd isoform was used for crystallization assays.

### 4.2. Crystallization

The lyophilized CBd was dissolved in 20 mM Tris (pH 7.4), 300 mM NaCl and 0.02% DDM, with a final protein concentration of 4.2 mg/mL. Initial crystallization conditions were screened at the Crystallography Platform of the Institut Pasteur [46] using MemGold screen kits (Molecular Dimensions), and gave small crystals after 8–10 days. Optimization was pursued on 24-well Linbro plates at 4 °C using the hanging-drop vapor-diffusion method. In the drop, 1 µL of crystallization buffer was mixed with 1 µL of CBd, giving a final protein concentration of 2.1 mg/mL. The best crystals were obtained with a solution containing 21% (*v*/*v*) PEG 400, 0.1 M NaCl, 0.18 M sodium acetate, and 0.1 M Tris (pH 8.0). The crystals grew within seven weeks at 4 °C.

### 4.3. Data Collection and Refinement

Single crystals of CBd were rinsed briefly in solutions containing the crystallization buffer with 30% (*v*/*v*) PEG 400 as cryoprotectant and flash-cooled in liquid nitrogen for data collection at cryogenic temperature. X-ray diffraction data were measured on beamline Proxima-1 at synchrotron SOLEIL (Saint-Aubin, France). The diffraction images were integrated with XDS [47] and crystallographic calculations carried out with the ccp4 program suite [48]. The structure was solved by molecular replacement with the Phaser [49] using crotoxin isoform CBb (pdb 3R0L, polypeptide chain D) as a template. The structure was refined with Buster [50] and manual adjustments were made to the model with Coot [51]. Atomic coordinates and structure factors have been deposited in the Protein Data Bank (pdb) with accession code 6TMY.

### 4.4. Bioinformatics Analysis of Sequences

For bioinformatics analysis amino acid sequences of *Crotalus durissus terrificus* CB isoforms deposited in GenBank/National Center for Biotechnology Information (NCBI) data base (P24027.1 (CBa_2_), P0CG56.1 (CBb), P62022.1 (CBc)) were used. Multiple alignment analysis of the sequences was performed using CLC Sequence Viewer 6.8 software.

## 5. Conclusions

Snake venoms are an important source of biologically active molecules that, in addition to toxicity, can reveal a therapeutic potential. Detailed characterization of these proteins is crucial for the possibility of their use in medical and pharmacological applications. Here we presented the three-dimensional structure of CBd, the most abundant isoform of the basic PLA_2_ subunit of crotoxin. Comparison of its three-dimensional (3D) structure and quaternary organization, as well as amino acid sequence, with the three other CB isoforms indicated specific single residue variations that are important for the functional diversity of crotoxin complexes. We also described the functional sites of CB that allow for its interaction with other proteins. These functional sites were previously determined experimentally by biophysical and biochemical methods (protein-protein interaction using SPR, affinity binding studies and competition experiments, physiological and electrophysiological functional analysis, hydrogen-deuterium exchange mass spectrometry, molecular docking simulations and crystallographic studies). The binding interface of four complexes (CA_2_-CBb, CBc-FXa, CBb-∆F508NBD1) and CBd-CBd (this study), identified at the level of specific amino acid residues, significantly overlap. Determination of such interaction platforms may be useful for developing new anti-cystic fibrosis, as well as anticoagulant, therapeutics.

## Figures and Tables

**Table 1:** Crystallographic data and refinement statistics.

**Crystal Parameters**	
Space group	C2
Unit cell dimensions, Å	a = 149.63, b = 75.59, c = 109.01
Angles, °	90, 121.8, 90
Vm, Å^3^/Da	3.1
**Data Statistics**	
Resolution, Å	45.5–1.80 (1.83–1.80)
Unique reflections	99489 (4708)
Multiplicity	4.7 (4.0)
Rmerge	0.078 (0.834)
Rpim	0.061 (0.681)
Completeness, %	98.9 (97.0)
<I/sigma(I)>	9.6 (1.5)
CC(1/2)	0.997 (0.508)
**Refinement**	
Resolution, Å	47.5–1.80 (1.81–1.80)
R-value (working set)	0.192 (0.261)
Rfree	0.214 (0.350)
Number of reflections	94480 (1890)
Number of atoms	
Protein	5879
Heterogen	149
Solvent	812
rms deviations from ideal	
Bond length, Å	0.010
Bond angles, °	0.88
Ramachandran plot	
Preferred regions, %	96.7
Allowed regions, %	3.3

Values in parentheses are for the highest resolution shell. Abbreviations: Rpim: precision-indicating merging R, CC(1/2): half-dataset correlation coefficient.

**Table 2:** Distinguishing residues in CB isoforms form two classes of crotoxin complexes.

aa Position *	Class I			Class II
	CBb	CBc	CBd	CBa_2_
**1**	His			Ser
**19**	Val	Ile	Ile	Val
**34**	Gln	Arg	Arg	Gln
**74**	Arg	Pro	Arg	Arg
**92**	Glu			Lys
**115**	Asn	Tyr	Tyr	Asn
**116**	Gly			Glu
**128**	Gly			Glu

* residues are numbered in accordance to the system proposed by Renetseder et al. [26].

## References

[1] Hendon R.A., Fraenkel-Conrat H. (1971). Biological roles of the two components of crotoxin. Proc. Natl. Acad. Sci. USA.

[2] Faure G., Bon C. (1987). Several isoforms of crotoxin are present in individual venoms from the South American rattlesnake Crotalus durissus terrificus. Toxicon.

[3] Faure G., Bon C. (1988). Crotoxin, a phospholipase A_2_ neurotoxin from the South American rattlesnake crotalus durissus terrificus: Purification of several isoforms and comparison of their molecular structure and of their biological activities. Biochemistry.

[4] Faure G., Guillaume J.L., Camoin L., Saliou B., Bon C. (1991). Multiplicity of acidic subunit isoforms of crotoxin, the phospholipase A_2_ neurotoxin from Crotalus durissus terrificus venom, results from posttranslational modifications. Biochemistry.

[5] Faure G., Harvey A.L., Thomson E., Saliou B., Radvanyi F., Bon C. (1993). Comparison of crotoxin isoforms reveals that stability of the complex plays a major role in its pharmacological action. Eur. J. Biochem..

[6] Hawgood B.J., Smith J.W. (1977). The mode of action at the mouse neuromuscular junction of the phospholipase A—Crotapotin complex isolated from venom of the South American rattlesnake. Br. J. Pharmacol..

[7] Krizaj I., Faure G., Gubensek F., Bon C. (1997). Neurotoxic phospholipases A_2_ ammodytoxin and crotoxin bind to distinct high-affinity protein acceptors in Torpedo marmorata electric organ. Biochemistry.

[8] Faure G., Copic A., Le Porrier S., Gubensek F., Bon C., Krizaj I. (2003). Crotoxin acceptor protein isolated from Torpedo electric organ: Binding properties to crotoxin by surface plasmon resonance. Toxicon.

[9] Shimizu J.F., Pereira C.M., Bittar C., Batista M.N., Campos G.R.F., da Silva S., Cintra A.C.O., Zothner C., Harris M., Sampaio S.V. (2017). Multiple effects of toxins isolated from Crotalus durissus terrificus on the hepatitis C virus life cycle. PLoS ONE.

[10] Cura J.E., Blanzaco D.P., Brisson C., Cura M.A., Cabrol R., Larrateguy L., Mendez C., Sechi J.C., Silveira J.S., Theiller E. (2002). Phase I and pharmacokinetics study of crotoxin (cytotoxic PLA_2_, NSC-624244) in patients with advanced cancer. Clin. Cancer Res..

[11] Wang J., Qin X., Zhang Z., Chen M., Wang Y., Gao B. (2014). Crotoxin suppresses the tumorigenic properties and enhances the antitumor activity of Iressa^®^ (gefinitib) in human lung adenocarcinoma SPCA-1 cells. Mol. Med. Rep..

[12] Yan C., Yang Y., Qin Z., Gu Z., Reid P., Liang Z. (2007). Autophagy is involved in cytotoxic effects of crotoxin in human breast cancer cell line MCF-7 cells. Acta Pharmacol. Sin..

[13] Sampaio S.C., Rangel-Santos A.C., Peres C.M., Curi R., Cury Y. (2005). Inhibitory effect of phospholipase A_2_ isolated from crotalus durissus terrificus venom on macrophage function. Toxicon.

[14] Zambelli V.O., Sampaio S.C., Sudo-Hayashi L.S., Greco K., Britto L.R.G., Alves A.S., Zychar B.C., Gonçalves L.R.C., Spadacci-Morena D.D., Otton R. (2008). Crotoxin alters lymphocyte distribution in rats: Involvement of adhesion molecules and lipoxygenase-derived mediators. Toxicon.

[15] Freitas A.P., Favoretto B.C., Clissa P.B., Sampaio S.C., Faquim-Mauro E.L. (2018). Crotoxin isolated from crotalus durissus terrificus venom modulates the functional activity of dendritic cells via formyl peptide receptors. J. Immunol. Res..

[16] Zhang H.-L., Han R., Chen Z.-X., Chen B.-W., Gu Z.-L., Reid P.F., Raymond L.N., Qin Z.-H. (2006). Opiate and acetylcholine-independent analgesic actions of crotoxin isolated from crotalus durissus terrificus venom. Toxicon.

[17] da Silva Lomeo R., de Faria Gonçalves A.P., da Silva C.N., de Paula A.T., Santos D.O.C., Fortes-Dias C.L., Gomes D.A., de Lima M.E. (2014). Crotoxin from crotalus durissus terrificus snake venom induces the release of glutamate from cerebrocortical synaptosomes via N and P/Q calcium channels. Toxicon.

[18] Krizaj I., Faure G., Gubensek F., Bon C. (1996). Re-examination of crotoxin-membrane interactions. Toxicon.

[19] Faure G., Gowda V.T., Maroun R.C. (2007). Characterization of a human coagulation factor Xa-binding site on Viperidae snake venom phospholipases A_2_ by affinity binding studies and molecular bioinformatics. BMC Struct. Biol..

[20] Faure G., Bakouh N., Lourdel S., Odolczyk N., Premchandar A., Servel N., Hatton A., Ostrowski M.K., Xu H., Saul F.A. (2016). Rattlesnake phospholipase A_2_ increases CFTR-chloride channel current and corrects ∆F508CFTR dysfunction: Impact in cystic fibrosis. J. Mol. Biol..

[21] Ostrowski M., Porowinska D., Prochnicki T., Prevost M., Raynal B., Baron B., Sauguet L., Corringer P.-J., Faure G. (2016). Neurotoxic phospholipase A_2_ from rattlesnake as a new ligand and new regulator of prokaryotic receptor GLIC (proton-gated ion channel from G. violaceus). Toxicon.

[22] Fortes-Dias C.L., Lin Y., Ewell J., Diniz C.R., Liu T.Y. (1994). A phospholipase A_2_ inhibitor from the plasma of the South American rattlesnake (Crotalus durissus terrificus). Protein structure, genomic structure, and mechanism of action. J. Biol. Chem..

[23] Perales J., Villela C., Domont G.B., Choumet V., Saliou B., Moussatché H., Bon C., Faure G. (1995). Molecular structure and mechanism of action of the crotoxin inhibitor from crotalus durissus terrificus serum. Eur. J. Biochem..

[24] Faure G. (2000). Natural inhibitors of toxic phospholipases A_2_. Biochimie.

[25] Faure G., Porowinska D., Saul F., Gopalakrishnakone P., Cruz L.J., Luo S. (2017). Crotoxin from crotalus durissus terrificus and crotoxin-related proteins: Structure and function relationship. Toxins and Drug Discovery.

[26] Renetseder R., Brunie S., Dijkstra B.W., Drenth J., Sigler P.B. (1985). A comparison of the crystal structures of phospholipase A_2_ from bovine pancreas and Crotalus atrox venom. J. Biol. Chem..

[27] Faure G., Xu H., Saul F.A. (2011). Crystal structure of crotoxin reveals key residues involved in the stability and toxicity of this potent heterodimeric β-neurotoxin. J. Mol. Biol..

[28] Marchi-Salvador D.P., Corrêa L.C., Magro A.J., Oliveira C.Z., Soares A.M., Fontes M.R.M. (2008). Insights into the role of oligomeric state on the biological activities of crotoxin: Crystal structure of a tetrameric phospholipase A_2_ formed by two isoforms of crotoxin B from Crotalus durissus terrificus venom. Proteins.

[29] Scott D.L., White S.P., Otwinowski Z., Yuan W., Gelb M.H., Sigler P.B. (1990). Interfacial catalysis: The mechanism of phospholipase A_2_. Science.

[30] Krissinel E., Henrick K. (2007). Inference of macromolecular assemblies from crystalline state. J. Mol. Biol..

[31] Snitko Y., Koduri R.S., Han S.K., Othman R., Baker S.F., Molini B.J., Wilton D.C., Gelb M.H., Cho W. (1997). Mapping the interfacial binding surface of human secretory group IIa phospholipase A_2_. Biochemistry.

[32] Zambelli V.O., Picolo G., Fernandes C.A.H., Fontes M.R.M., Cury Y. (2017). Secreted phospholipases A_2_ from animal venoms in pain and analgesia. Toxins.

[33] Fortes-Dias C.L., dos Santos R.M.M., Magro A.J., de Mattos Fontes M.R., Chávez-Olórtegui C., Granier C. (2009). Identification of continuous interaction sites in PLA_2_-based protein complexes by peptide arrays. Biochimie.

[34] Faure G., Saul F. (2011). Structural and functional characterization of anticoagulant, FXa-binding Viperidae snake venom phospholipases A_2_. Acta Chim. Slov..

[35] Faure G., Xu H., Saul F., Kini R.M., Clemetson K.J., Markland F.S., McLane M.A., Morita T. (2010). Anticoagulant phospholipases A_2_ which bind to the specific soluble receptor coagulation factor Xa. Toxins and Hemostasis: From Bench to Bedside.

[36] Ostrowski M., Žnidaršič P.P., Raynal B., Saul F., Faure G. (2014). Human coagulation factor Xa prevents oligomerization of anti-coagulant phospholipases A_2_. Toxin Rev..

[37] Petan T., Križaj I., Gelb M.H., Pungerčar J. (2005). Ammodytoxins, potent presynaptic neurotoxins, are also highly efficient phospholipase A_2_ enzymes. Biochemistry.

[38] Saul F.A., Prijatelj-Znidarsic P., Vulliez-le Normand B., Villette B., Raynal B., Pungercar J., Krizaj I., Faure G. (2010). Comparative structural studies of two natural isoforms of ammodytoxin, phospholipases A_2_ from Vipera ammodytes ammodytes which differ in neurotoxicity and anticoagulant activity. J. Struct. Biol..

[39] Radvanyi F.R., Bon C. (1982). Catalytic activity and reactivity with p-bromophenacyl bromide of the phospholipase subunit of crotoxin. Influence of dimerization and association with the noncatalytic subunit. J. Biol. Chem..

[40] Faure G., Saul F. (2012). Crystallographic characterization of functional sites of crotoxin and ammodytoxin, potent β-neurotoxins from Viperidae venom. Toxicon.

[41] Šribar J., Kovačič L., Oberčkal J., Ivanušec A., Petan T., Fox J.W., Križaj I. (2019). The neurotoxic secreted phospholipase A_2_ from the Vipera a. ammodytes venom targets cytochrome c oxidase in neuronal mitochondria. Sci. Rep..

[42] Mounier C.M., Luchetta P., Lecut C., Koduri R.S., Faure G., Lambeau G., Valentin E., Singer A., Ghomashchi F., Béguin S. (2000). Basic residues of human group IIA phospholipase A_2_ are important for binding to factor Xa and prothrombinase inhibition comparison with other mammalian secreted phospholipases A_2_. Eur. J. Biochem..

[43] Vadas P., Browning J., Edelson J., Pruzanski W. (1993). Extracellular phospholipase A_2_ expression and inflammation: The relationship with associated disease states. J. Lipid Mediat..

[44] Faure G., Saul F. Caractéristiques structurales et fonctionnelles de deuxβ-neurotoxines: L’ammodytoxine et la crotoxine. Proceedings of the Toxines et Fonctions Cholinergiques Neuronales et non Neuronales.

[45] Faure G., Choumet V., Bouchier C., Camoin L., Guillaume J.L., Monegier B., Vuilhorgne M., Bon C. (1994). The origin of the diversity of crotoxin isoforms in the venom of Crotalus durissus terrificus. Eur. J. Biochem..

[46] Weber P., Pissis C., Navaza R., Mechaly A.E., Saul F., Alzari P.M., Haouz A. (2019). High-throughput crystallization pipeline at the crystallography core facility of the institut pasteur. Molecules.

[47] Kabsch W. (2010). Integration, scaling, space-group assignment and post-refinement. Acta Crystallogr. D Biol. Crystallogr..

[48] Winn M.D., Ballard C.C., Cowtan K.D., Dodson E.J., Emsley P., Evans P.R., Keegan R.M., Krissinel E.B., Leslie A.G.W., McCoy A. (2011). Overview of the CCP4 suite and current developments. Acta Crystallogr. D Biol. Crystallogr..

[49] McCoy A.J., Grosse-Kunstleve R.W., Adams P.D., Winn M.D., Storoni L.C., Read R.J. (2007). Phaser crystallographic software. J Appl. Crystallogr..

[50] Bricogne G., Blanc E., Brandl M., Flensburg C., Keller P., Paciorek P., Roversi P., Sharff A., Smart O., Vonrhein C. (2010). BUSTER.

[51] Emsley P., Lohkamp B., Scott W.G., Cowtan K. (2010). Features and development of Coot. Acta Crystallogr. D Biol. Crystallogr..

